# Supplementary material for: Cationic Polystyrene-Based Hydrogels as Efficient Adsorbents to Remove Methyl Orange and Fluorescein Dye Pollutants from Industrial Wastewater
Source: Int J Mol Sci. 2023 Feb 2;24(3):2948. doi: 10.3390/ijms24032948 (PMC9918298; doi:10.3390/ijms24032948)
Supplement: Supplementary file 1 [file ijms-24-02948-s001.zip › ijms-2164105-Supplementary.pdf]

Article

Cationic Polystyrene-Based Hydrogels as Efficient Adsorbents to Remove Methyl Orange and Fluorescein Dyes Pollutants from Industrial Wastewater

Silvana Alfei *, Federica Grasso, Valentina Orlandi, Eleonora Russo, Raffaella Boggia and Guendalina Zuccari *

Department of Pharmacy, University of Genoa, Viale Cembrano, 16148 Genoa, Italy

* Correspondence: alfei@difar.unige.it; Tel.: +39-010-355-2296 (S.A.); zuccari@difar.unige.it (G.Z.)

Section S1. Analysis of the miscrostructure of R1.

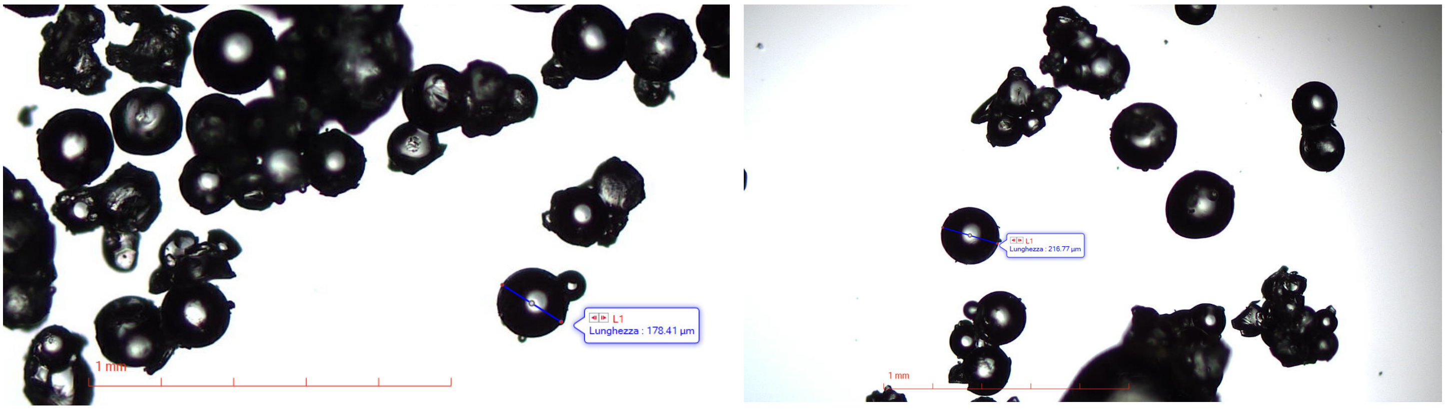

(a) (b)

Figure S1. Optic images (using a 4 × objective) of R1 showing microdimensioned particle of 178 µm (**a**) and 217 µm (**b**).

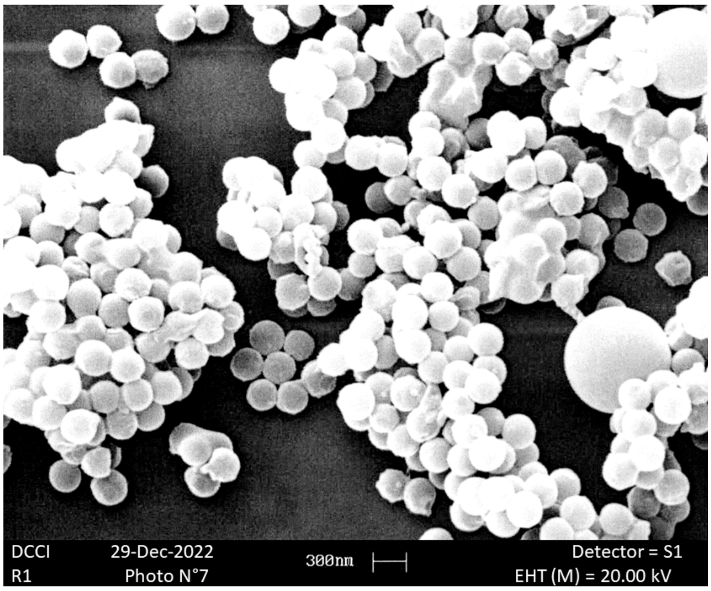

Figure S2. SEM image of resin R1.

Section S2. Preparation of R1HG and R2HG.

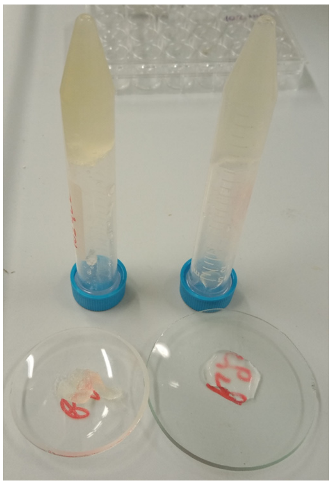

Figure S3. Appearance of R1HG (left side) and R2HG (right side) at their equilibrium degree of swelling (EDS).

Section S3. Weight Loss Experiments.

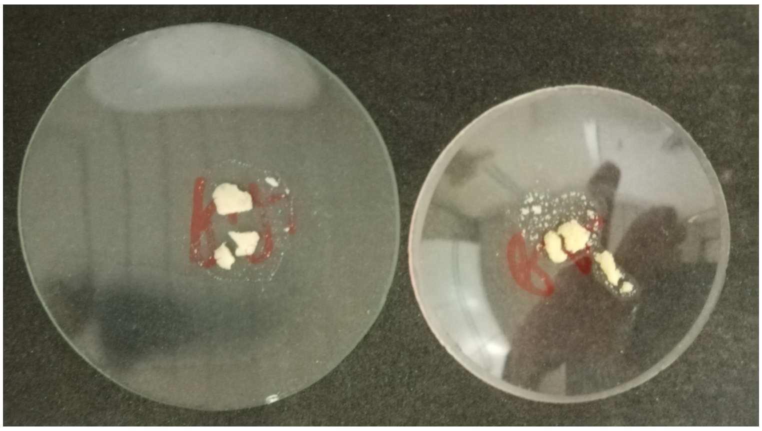

Figure S4. Appearance of fully dried D-R1HG (right side) and D-R2HG (right side) when a constant weight was reached.

Section S4. Rehological Experiments.

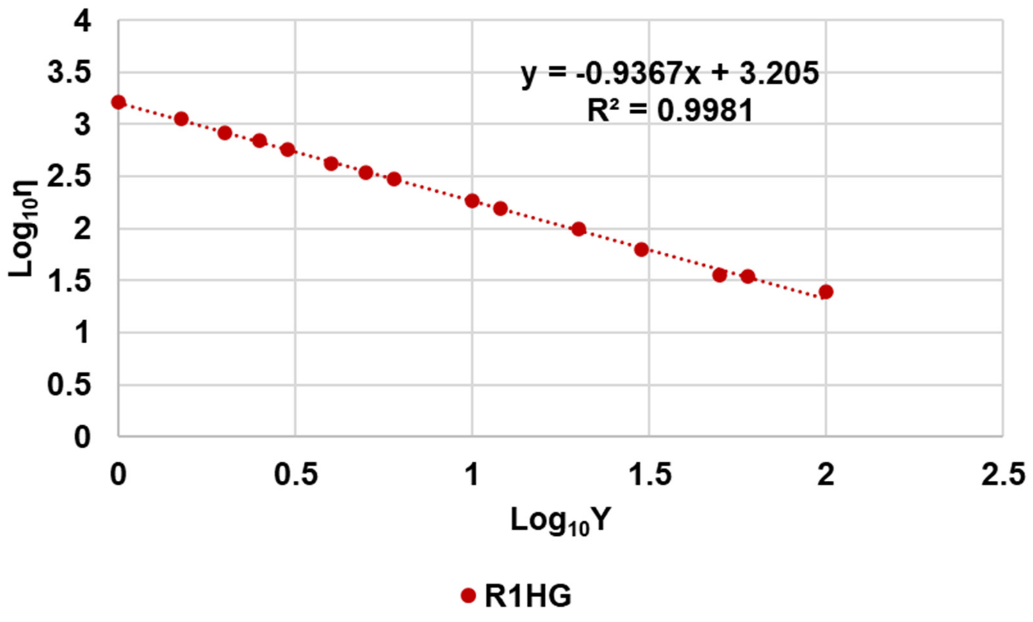

Figure S5. Linear relationship between Log η and Log γ of R1HG to determine n.

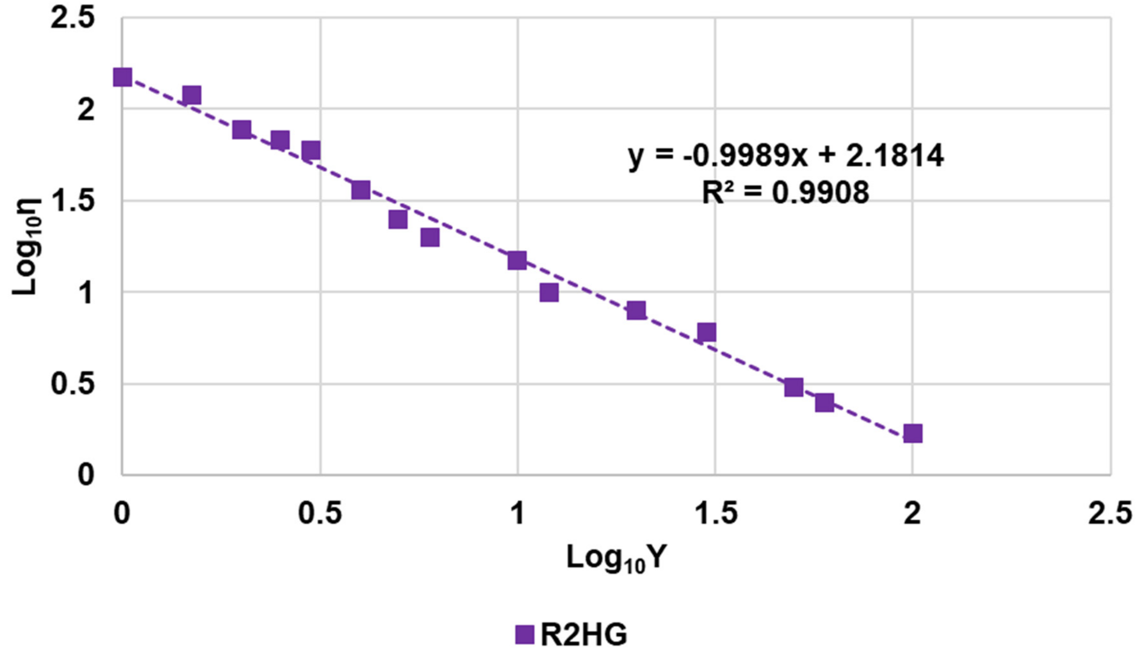

Figure S6. Linear relationship between Log η and Log γ of R2HG to determine n.

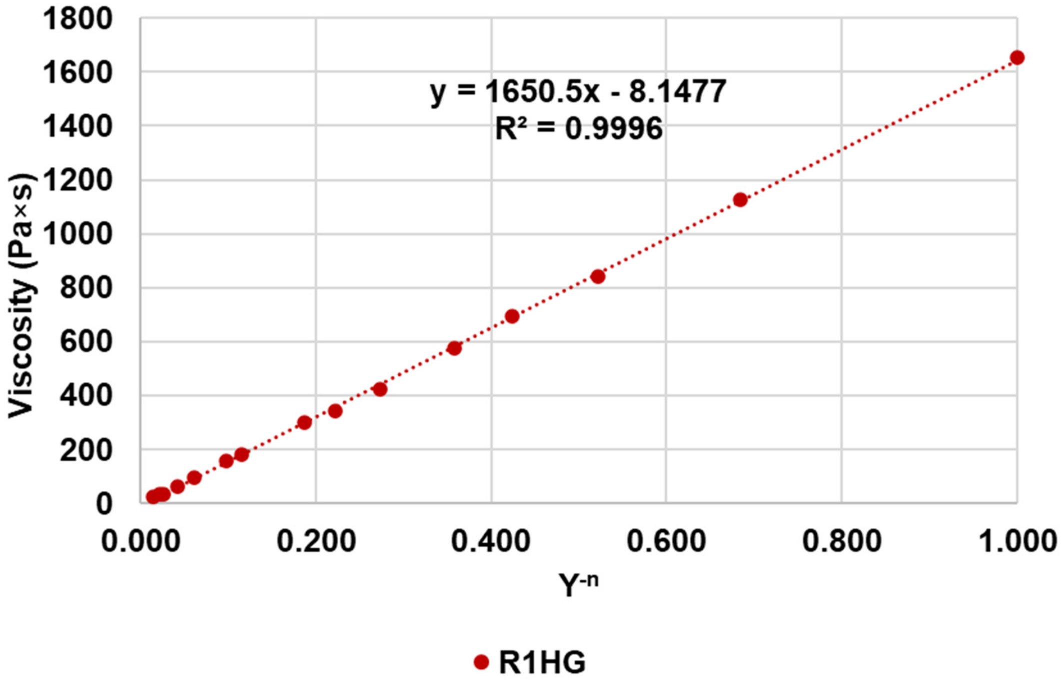

Figure S7. Linear relationship between η and γ^{-n} of R1HG to determine η∞.

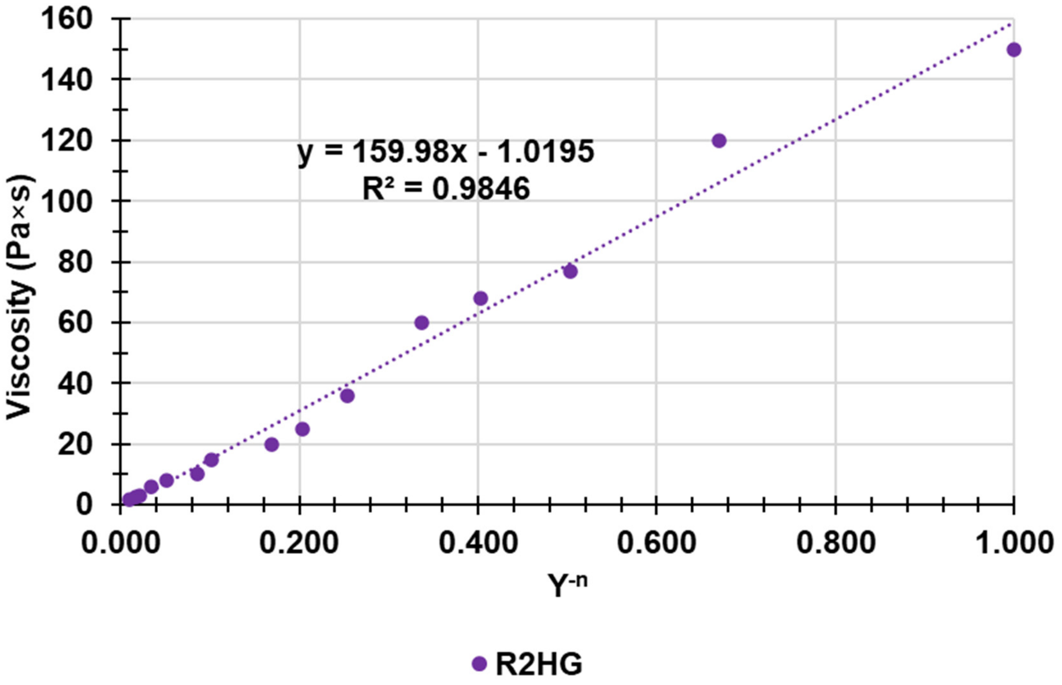

Figure S8. Linear relationship between η and γ^{-n} of R2HG to determine η∞.

Section S5. Dyes Removal Experiments using R1HG and R2HG.

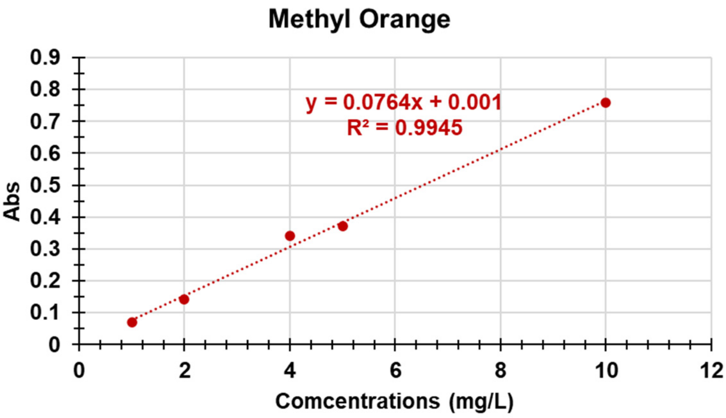

(**a**)

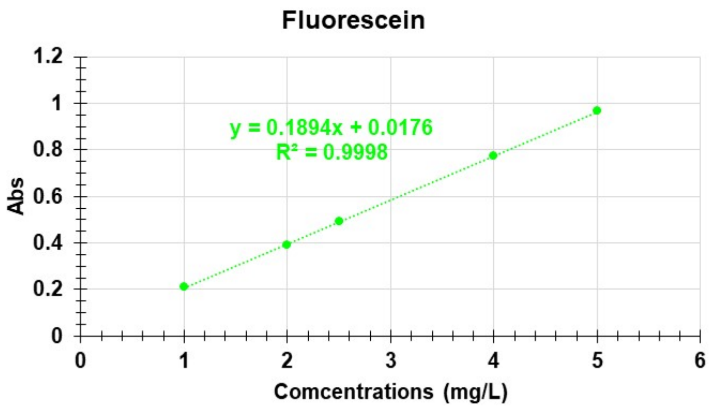

(**b**)

Figure S9. Linear calibration models for MO (**a**) and F (**b**). Error bars were not detectable.

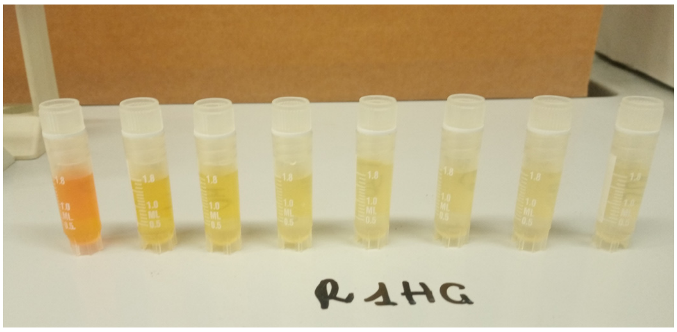

(**a**)

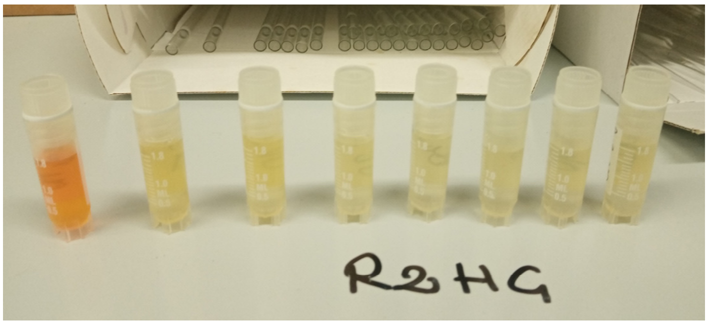

(**b**)

Figure S10. Appearance of the not diluted MO solutions at time T0 (untreated solutions, vials 1 on the left) and during treatment with R1 (**a**) and R2 (**b**) after 5, 10, 20, 30, 60, 90 and 120 minutes (vials 2-8 from the left to right).

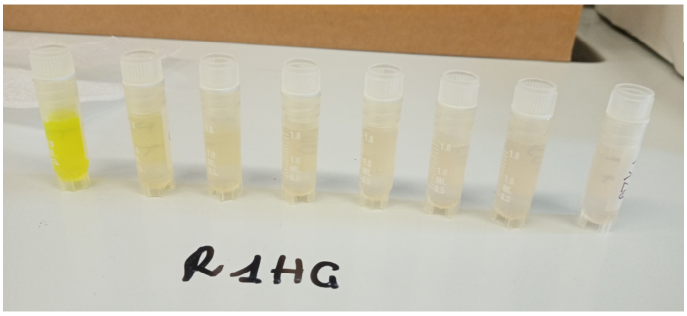

(**a**)

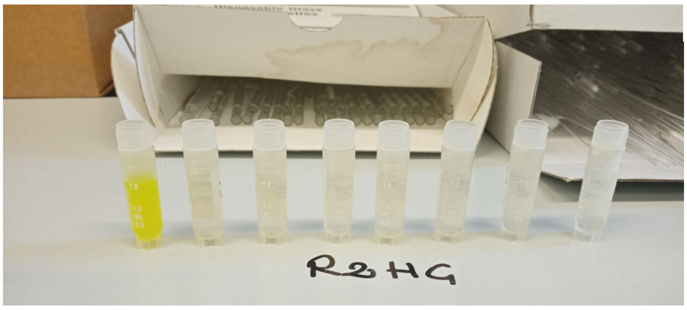

(**b**)

Figure S11. Appearance of the not diluted F solutions at time T0 (untreated solutions, vials 1 on the left) and during treatment with R1 (**a**) and R2 (**b**) after 5, 10, 20, 30, 60, 90 and 120 minutes (vials 2-8 from the left to right).

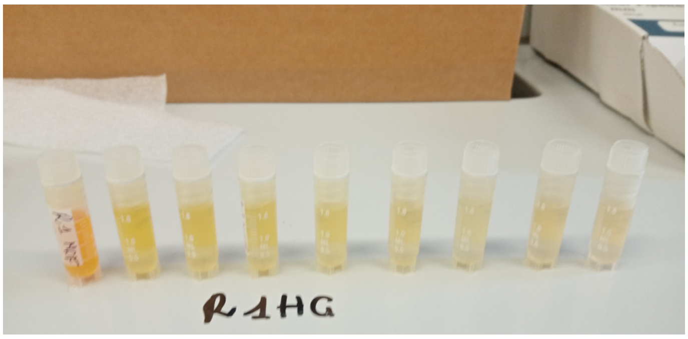

(**a**)

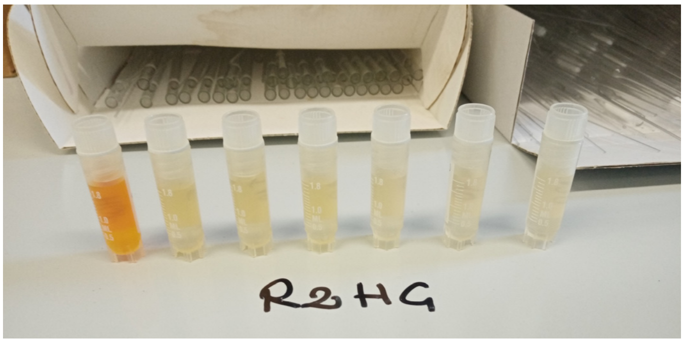

(**b**)

Figure S12. Appearance of not diluted MOF solutions at time T0 (untreated solutions, vials 1 on the left) and during treatment with R1 (**a**) and R2 (**b**) after 5, 10, 20, 30, 60, 90 and 120 minutes (vials 2-8 from the left to right).

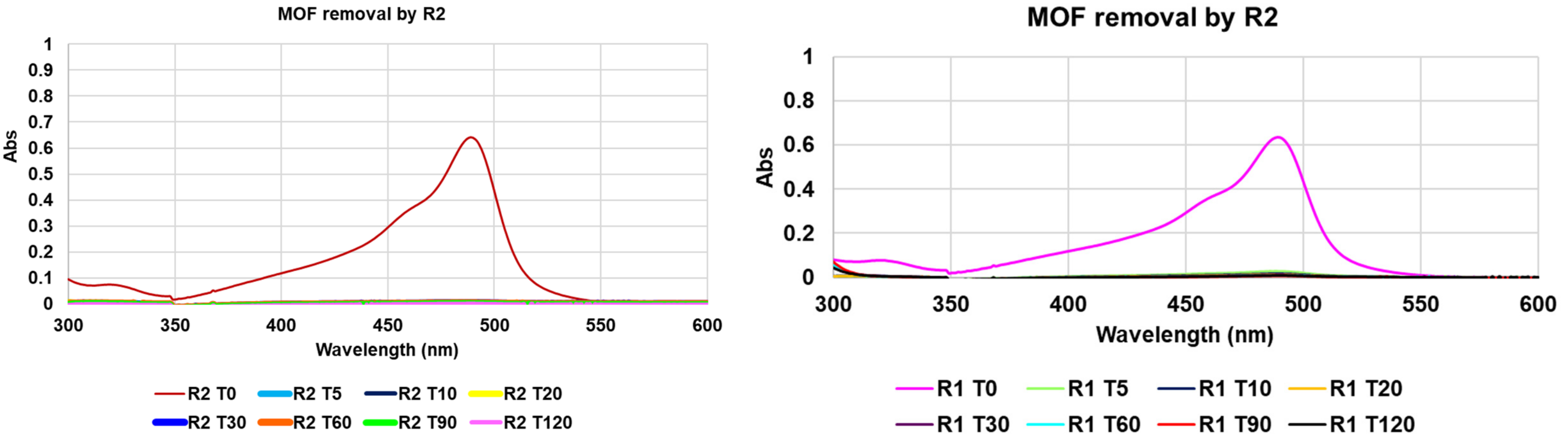

(a) (b)

Figure S13. UV-Vis spectra of MOF solutions at time T0 (lines red and fuchsia) and during treatment with resins R2 (**a**) and R1 (**b**).

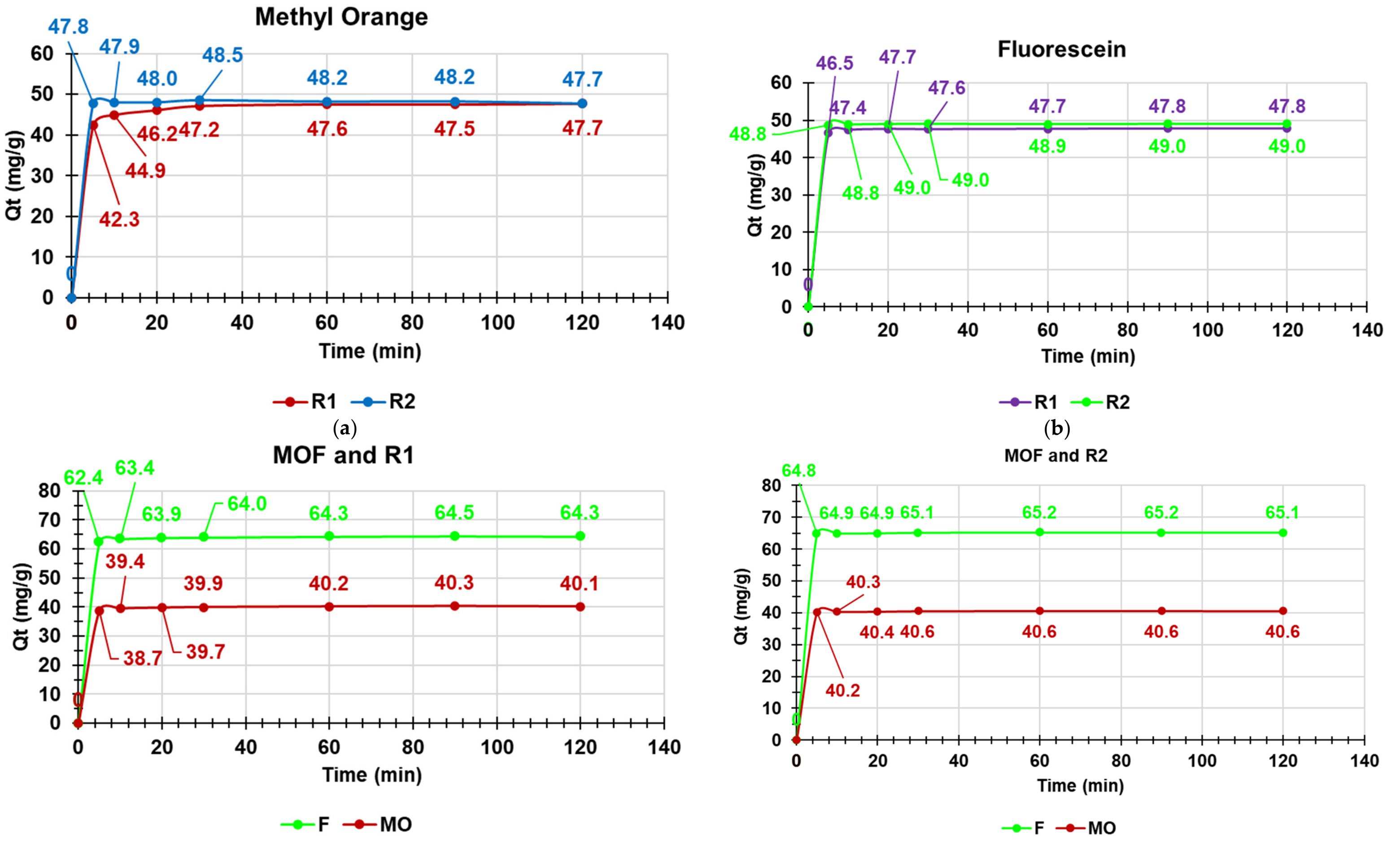

(c) (d)

Figure S14. Time-dependent adsorption (mg/g) of MO (**a**), F (**b**) on resins R1 and R2 (pH = 7.5; room temperature (r.t). Time-dependent adsorption (mg/g) of MO and F in MOF on resin R1 (**c**) and R2 (**d**) (pH = 7.5; r.t). F initial concentrations were 48.7 mg/L (R1), 49.4 mg/L (R2), 64.6 mg/L in MOF solution treated with R1, 64.9 mg/L in MOF solution treated with R2. MO initial concentrations were 50.9 mg/L (R1), 50.8 mg/L (R2), 39.6 mg/L in MOF solution treated with R1, 39.7 mg/L in MOF solution treated with R2.

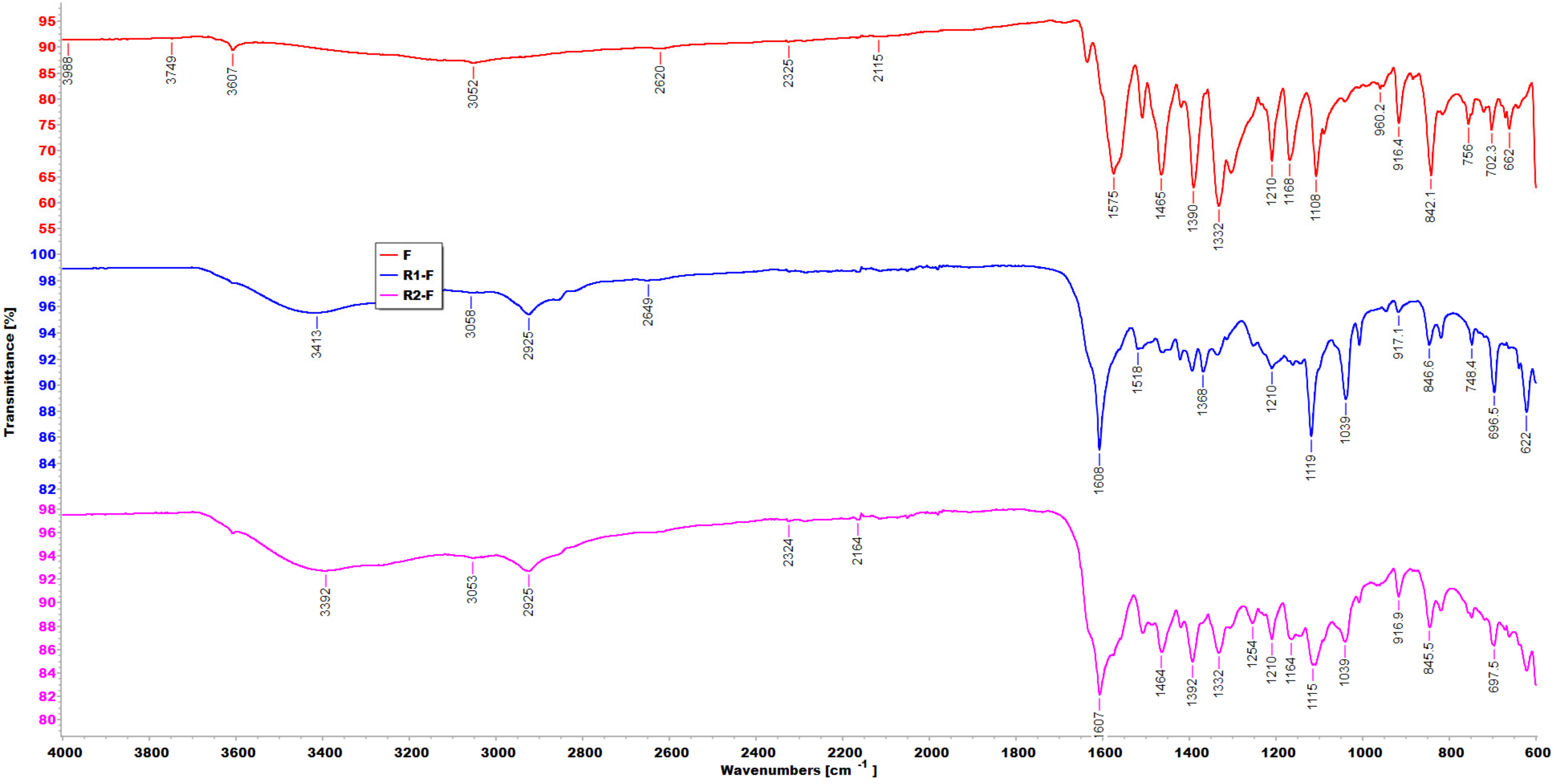

F
R1-F
R2-F
Transmittance [%]
Wavenumbers [cm -1]
3988
3749
3607
3052
2620
2325
2115
1575
1465
1390
1332
1210
1168
1108
960.2
916.4
842.1
756
702.3
662
3413
3058
2925
2649
1608
1518
1368
1210
1119
1039
917.1
846.6
748.4
696.5
622
3392
3053
2925
2324
2164
1607
1464
1392
1332
1254
1210
1164
1115
1039
916.9
845.5
697.5

(a)

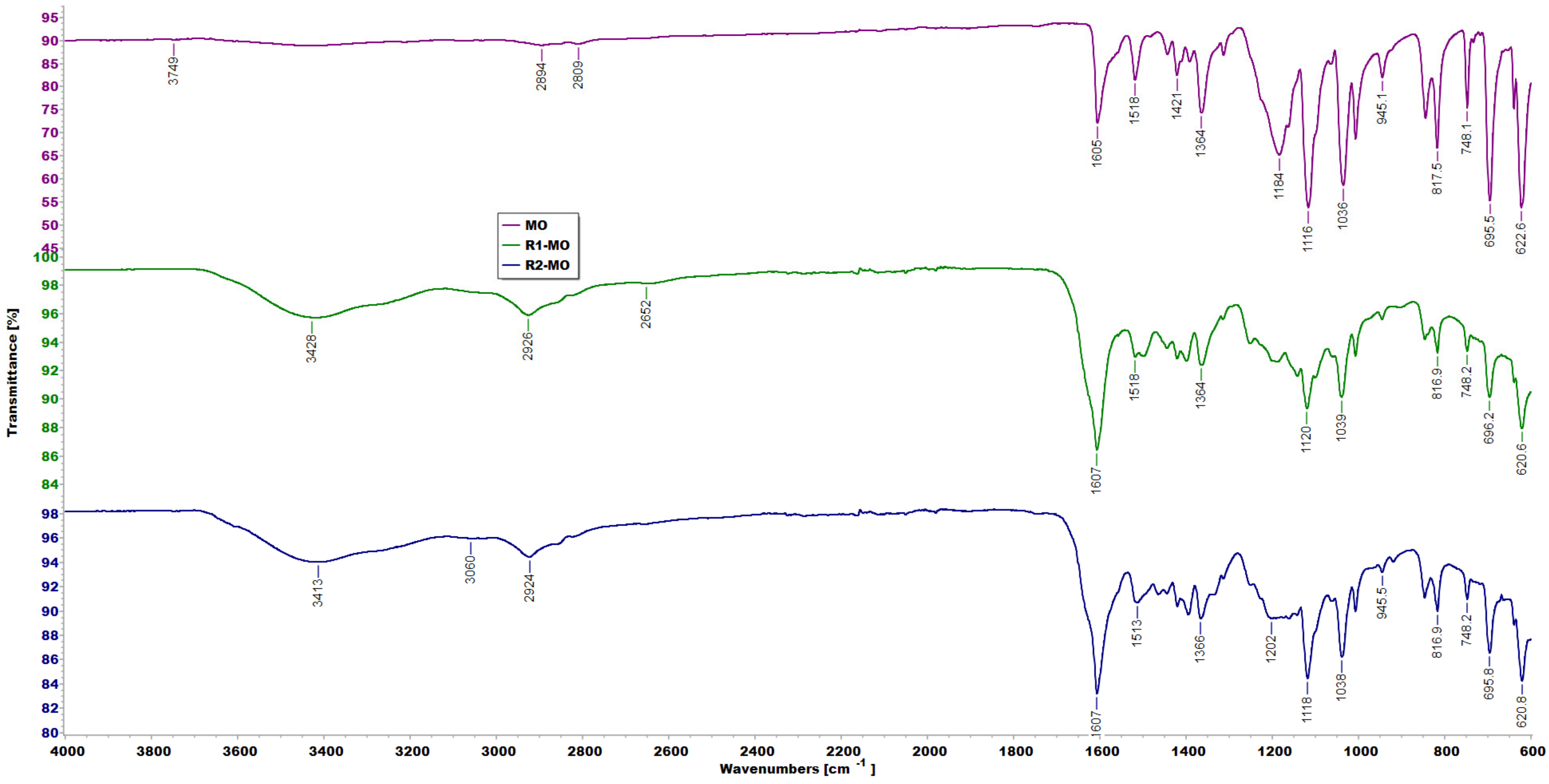

MO
R1-MO
R2-MO
Transmittance [%]
Wavenumbers [cm -1]
3749
2894
2809
1605
1518
1421
1364
1184
1116
1036
945.1
817.5
748.1
695.5
622.6
3428
2926
2652
1607
1518
1364
1120
1039
816.9
748.2
696.2
620.6
3413
3060
2924
1607
1513
1366
1202
1118
1038
945.5
816.9
748.2
695.8
620.8

(**b**)

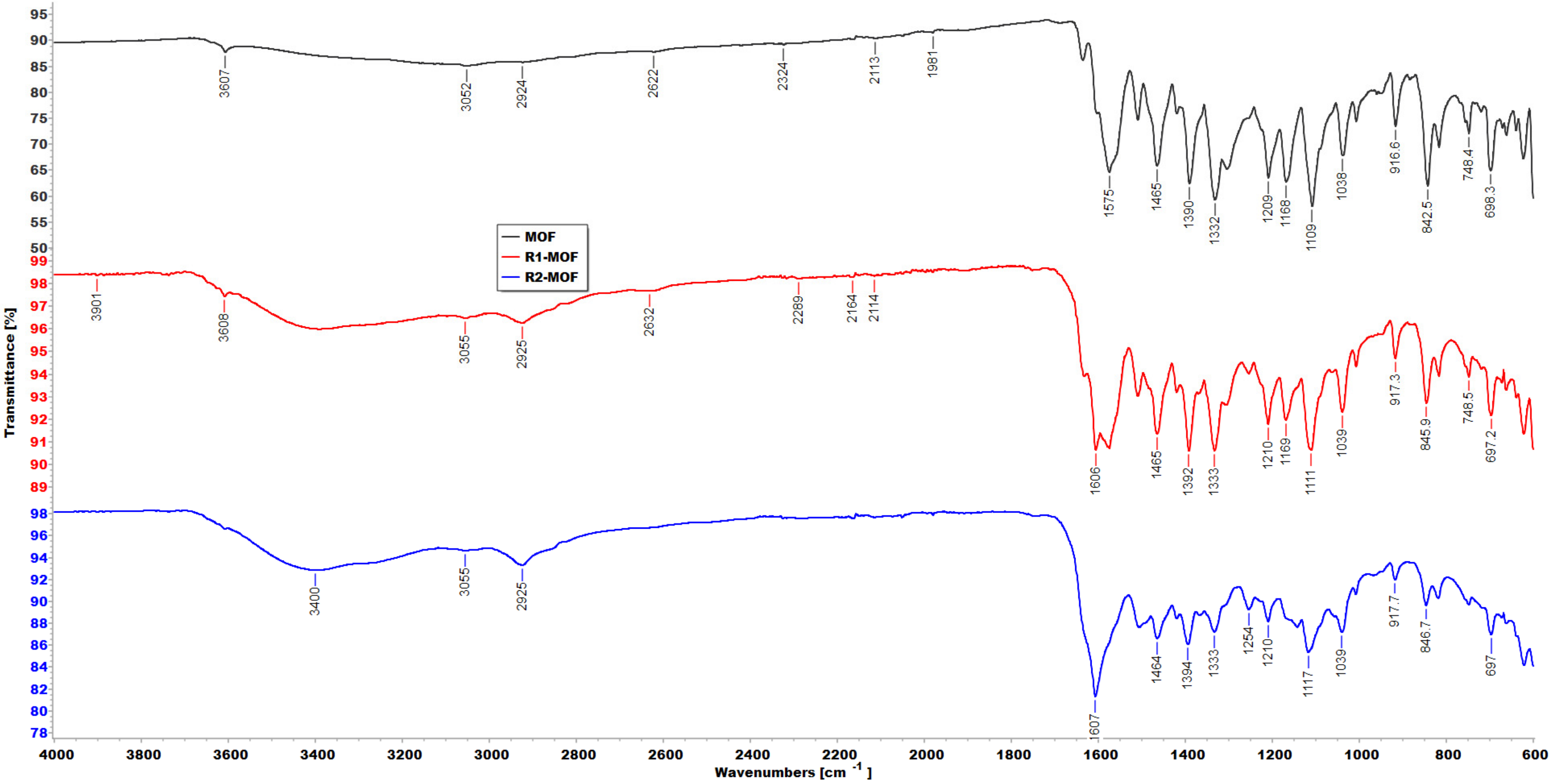

(**c**)

(**c**)

Figure S15. ATR-FTIR spectra of F (red line), R1 after absorption of F (blue line) and R2 after absorption of F (fuchsia line) (**a**), MO (purple line), R1 after absorption of MO (dark green line) and R2 after absorption of MO (dark blue line) (**b**), MOF (black line), R1 after absorption of MOF (red line) and R2 after absorption of MOF (blue line) (**c**).

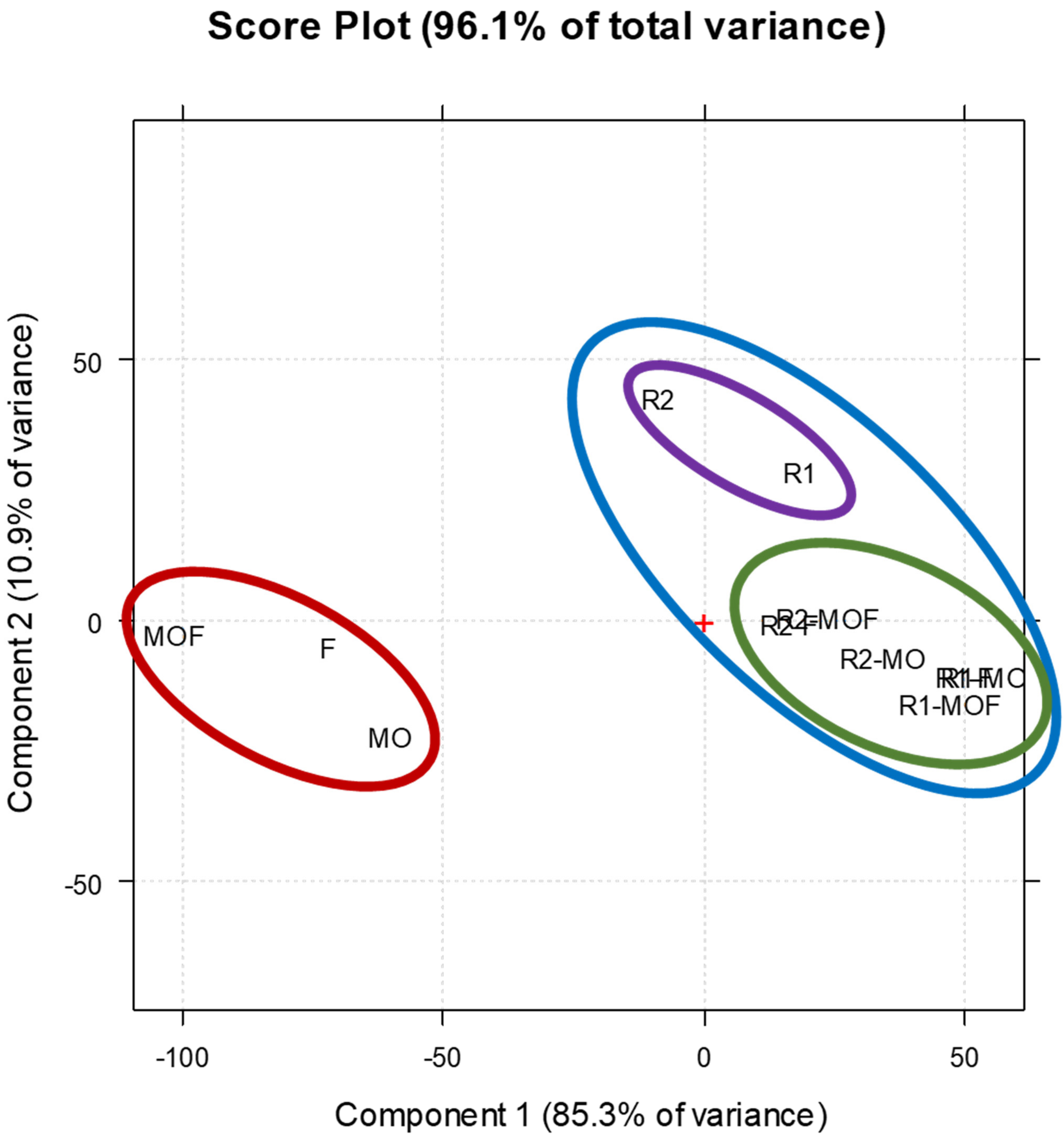

Figure S16. Results of PCA carried out on the matrix collecting data of spectra in Figure S15, plus those of R1 and R2 as score plot of PC1 vs. PC2.

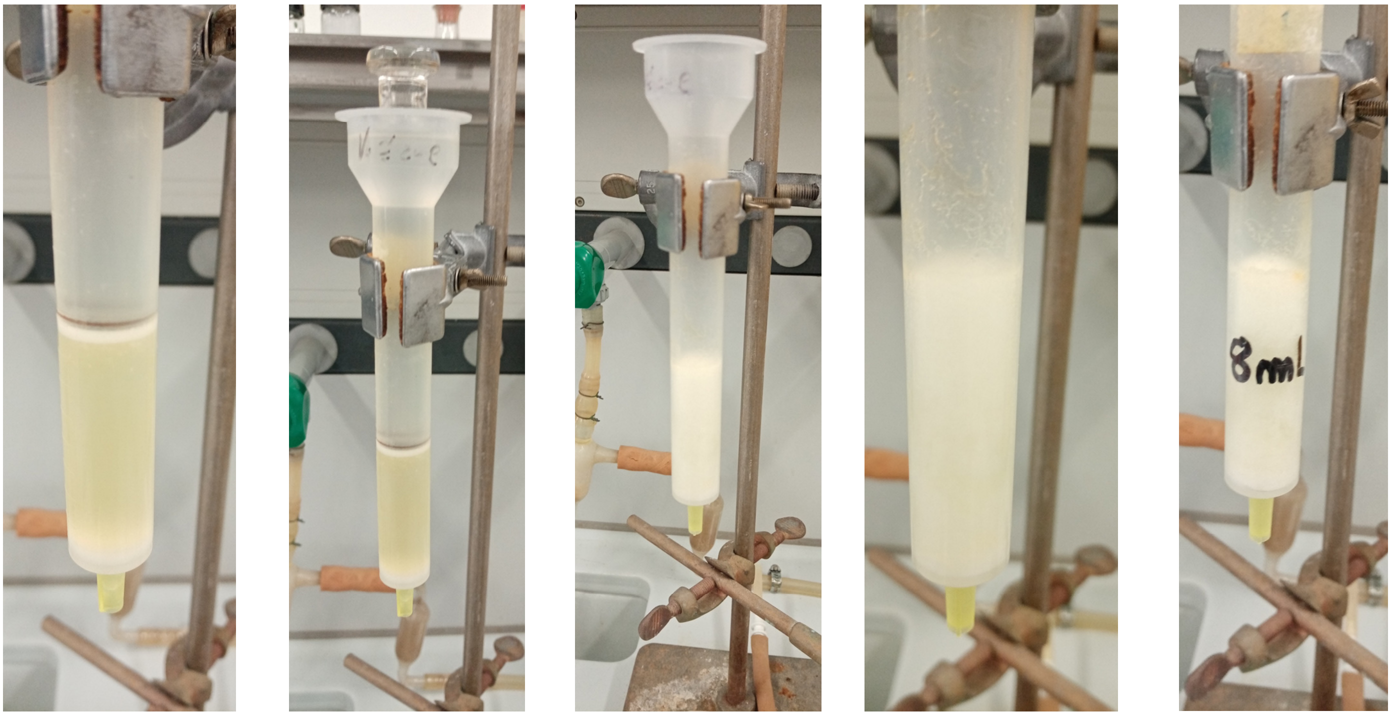

(a) (b) (c) (d) (e)

Figure S17. Typical column loaded with hydrogels. R1HG (a, **b**), R2HG (**c**, **d**); volume of columns (**e**).

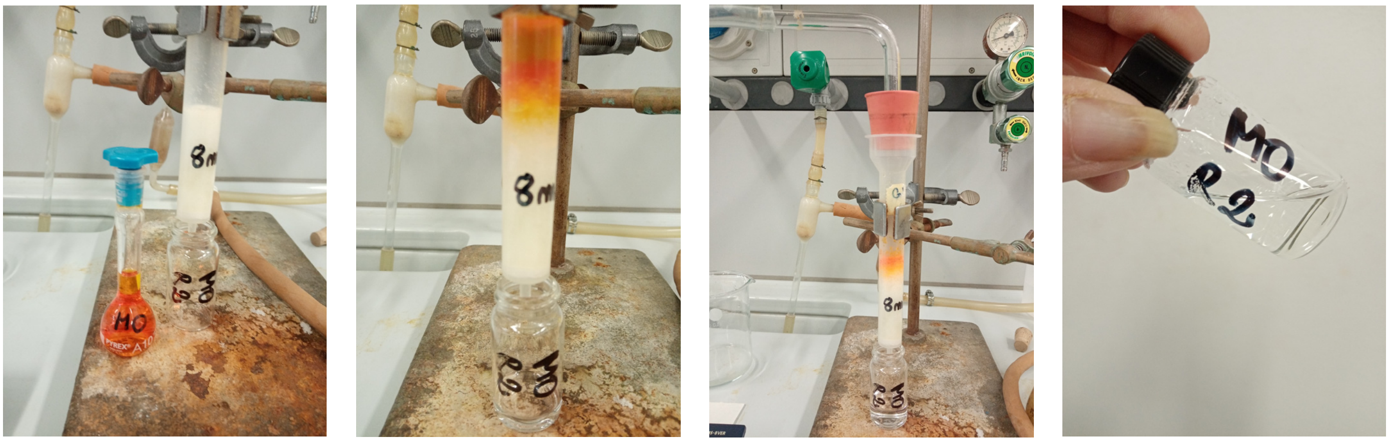

(a) (b) (c) (d)

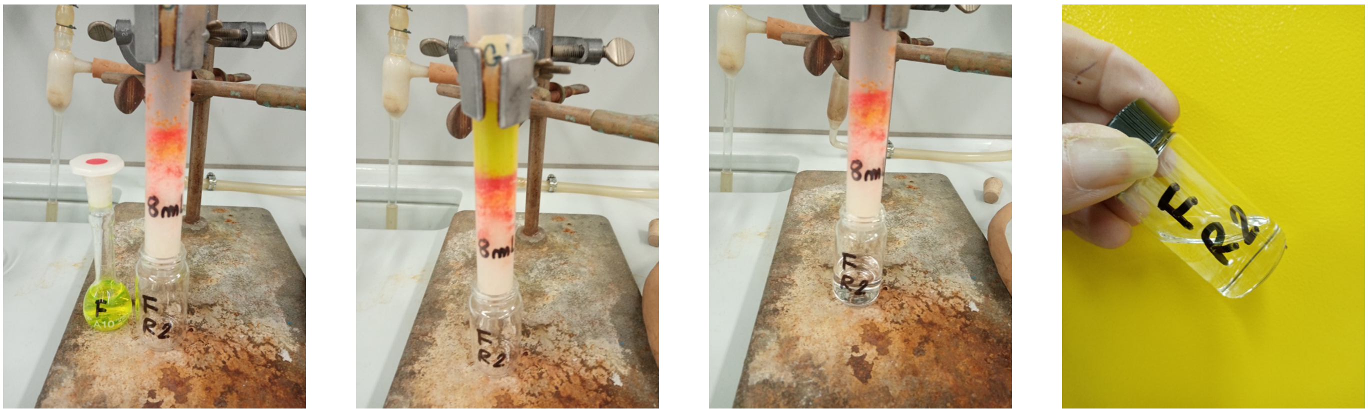

(e) (f) (g) (h)

Figure S18. Typical filtration experiment using R2 to prepare the gel column. MO (**a**) and F (**e**) (50 mg/L each one) were filtered on the column gel without refreshing it before the second filtration (**b**, **c**, **f**, **g**) and collecting the clear filtrates separately (**d**, **h**).
